# A flexible wide‐field FLIM endoscope utilising blue excitation light for label‐free contrast of tissue

**DOI:** 10.1002/jbio.201300203

**Published:** 2014-02-27

**Authors:** Hugh Sparks, Sean Warren, Joana Guedes, Nagisa Yoshida, Tze Choong Charn, Nadia Guerra, Taranjit Tatla, Christopher Dunsby, Paul French

**Affiliations:** ^1^ Photonics GroupPhysics DepartmentImperial College LondonLondon SW7 2AZUK; ^2^ Institute of Chemical BiologyImperial College LondonLondonSW7UK; ^3^ Cell and Molecular BiologyImperial College LondonLondonSW7UK; ^4^ Department of ENT‐Head &Neck SurgeryNorthwick Park HospitalHarrowHA1 3UJ

**Keywords:** FLIM, endoscope, autofluorescence, lifetime, fibre, flexible, lifetime, instrument

## Abstract

Fluorescence lifetime imaging (FLIM) has previously been shown to provide contrast between normal and diseased tissue. Here we present progress towards clinical and preclinical FLIM endoscopy of tissue autofluorescence, demonstrating a flexible wide‐field endoscope that utilised a low average power blue picosecond laser diode excitation source and was able to acquire ∼mm‐scale spatial maps of autofluorescence lifetimes from fresh ex vivo diseased human larynx biopsies in ∼8 seconds using an average excitation power of ∼0.5 mW at the specimen. To illustrate its potential for FLIM at higher acquisition rates, a higher power mode‐locked frequency doubled Ti:Sapphire laser was used to demonstrate FLIM of ex vivo mouse bowel at up to 2.5 Hz using 10 mW of average excitation power at the specimen. (© 2014 WILEY‐VCH Verlag GmbH &Co. KGaA, Weinheim)

Photo of ~2.3 mm diameter flexible endoscope distal tip.

## 1. Introduction

Fluorescence lifetime imaging (FLIM) [1, 2] of tissue autofluorescence has been shown to provide label-free contrast between different types and states of tissue that may help differentiate between normal and diseased human tissue, e.g. [3–8]. Building on the pioneering single-point clinical measurements of autofluorescence lifetime, e.g. [9, 10] and subsequent work recently reviewed in 2012 [11], clinical FLIM can combine lifetime contrast with morphological information to provide a direct comparison between different spatial regions – making it easier to spot differences with respect to "normal" tissue, e.g. for diagnostic screening and potentially enabling margins of diseased tissue to be identified. To date, however, there have been relatively few clinical FLIM studies, partly due to a lack of suitable instrumentation.

Intravital FLIM is being increasingly realised using multiphoton microscopy, for example in rodents, where cellular autofluorescence lifetimes have been correlated with changes in metabolism associated with cancer, e.g. [12], and in humans, e.g. [13] where studies of skin autofluorescence parameters including lifetime have been correlated with cancer [14, 15]. We are interested to apply FLIM to detect and monitor disease in internal organs such as the colon, for which flexible endoscope-based-instrumentation is required and where we believe that FLIM could complement current techniques such as white light endoscopy. Multiphoton excitation has also been utilised in endoscopes, as reviewed in 2005, [16] with implementations achieving minimally invasive devices of only a few mm diameter [17] that could facilitate optical biopsy in situ. For clinical applications such as diagnostic screening that involve rapidly surveying large areas of tissue, however, the sequential pixel scanning and expense of the required femtosecond laser technology make multiphoton excitation less practical than wide-field imaging with single-photon excitation. Confocal endomicroscopes have also been developed for FLIM [18, 19] and may find application for preclinical studies, e.g. using exogenous probes, but the laser scanning image acquisition is still impractical for rapid imaging of large areas of tissue. To image cm-scale fields of view in internal organs, we are therefore developing flexible wide-field FLIM endoscopes using coherent optical fibre bundles. In contrast to confocal or multiphoton imaging, wide-field FLIM lacks optical sectioning and therefore the fluorescence collected at each pixel can originate from (multiple) fluorophores within the penetration depth of the excitation radiation in the tissue. While this can compromise the measured decay profiles, the FLIM image will be dominated by the fluorophores nearer the surface and can still provide relative lifetime contrast across the field of view. This could make wide-field in vivo FLIM insufficiently quantitative for some applications but it could still serve to highlight potential anomalies, e.g. for diagnostic screening, to guide biopsies or to guide measurements using different modalities.

Wide-field FLIM endoscopy was first demonstrated ex vivo [3] using a frequency domain approach [1] to implement FLIM with a sinusoidally modulated argon ion excitation laser and was subsequently demonstrated using time-gated imaging with pulsed excitation at 80 MHz using a frequency-doubled mode-locked Ti:Sapphire laser at 415 nm [20]. The frequency domain system developed by Mizeret et al. could operate at up to video rate but with a field of view (FOV) of $32^2$ or $64^2$ pixels, while the first time-gated systems provided images of $>256^2$ pixels but required data acquisition times of the order of minutes. With improved fluorescence decay sampling strategies and optimised optical setups, full-field time-gated FLIM endoscopy of biological tissue with mode-locked Ti:Sapphire laser-based excitation has been demonstrated at 7.2 Hz in a rigid endoscope [21] and 5.5 Hz in a flexible endoscope [22]. The time-gated imaging and frequency domain approaches to FLIM both utilise image intensifiers that repetitively sample periodic fluorescence signals and should ultimately be able to achieve similar acquisition times and pixel counts.

Time-gated imaging has also been implemented using a Nitrogen laser at 337 nm to provide excitation with ~700 ps pulses at 20 Hz repetition rate [23]. This approach has been successfully translated to in vivo studies in hamster [24] and human [25] and has the advantage that the low duty cycle time-gated imaging enables FLIM in the presence of background room lights. Recently it has been extended to multispectral FLIM endoscopy [26] employing a frequency-tripled Q-switched Nd:YAG laser as the excitation source (wavelength 355 nm, repetition rate 100 kHz, pulse length <1 ns) and, using a two-gate rapid lifetime determination algorithm, has achieved FLIM at 2 frames per second in three spectral channels imaging a hamster cheek pouch model of cancer in vivo.

These results are encouraging but we believe that there remain several issues to address before wide-field FLIM endoscopy becomes sufficiently practical for routine clinical deployment. These include safety considerations associated with the use of u.v. excitation in vivo, the design of the endoscope to minimise background fluorescence and to realise an appropriate illumination of the FOV, the cost and complexity of the excitation source and the optimal detection and analysis of the autofluorescence signals. The optimum excitation wavelength for FLIM of autofluorescence to contrast diseased tissue will depend on the specific pathology but most studies have reported useful contrast with u.v. or blue radiation. In general, concerns associated with phototoxicity increase as the wavelength decreases into the ultraviolet and there are no established safe exposure limits for internal tissues. A further important issue is that u.v. and blue radiation can excite unwanted background fluorescence in optical components and this becomes increasingly severe with decreasing wavelength. For this reason we are exploring the use of blue excitation for FLIM endoscopy, noting that this can be provided by frequency doubled Ti:Sapphire lasers and also by gain switched diode lasers. The latter are particularly interesting because they are relatively compact and low cost compared to Ti:Sapphire lasers and are now commercially available with average powers of up to 20 mW.

We report here the development of a flexible wide-field FLIM endoscope with a FOV of 3 mm with which we have demonstrated the imaging of freshly resected human larynx samples using a gain-switched picosecond diode laser operating at 445 nm

Journal of BIOPHOTONICS

as the excitation source. For this particular excitation wavelength, we expect to be able to measure fluorescence signals from the metabolite, flavo adenine dinucleotide (FAD) [27] and the intercellular matrix components, collagen [28, 29] and elastin [27]. These endogenous fluorophores are considered to be potential biomarkers for disease progression [30]. This particular diode laser was limited to ~3 mW average power and so, to demonstrate rapid endoscopic FLIM with blue excitation, we also used a frequency-doubled Ti:Sapphire laser that was applied to excite ex vivo mouse bowel tissue. Wide-field illumination of the specimen was achieved using higher order mode propagation in a multimode optical fibre drawn from glass that presents low background fluorescence. Unfortunately, utilising multimode propagation in this excitation delivery fibre leads to temporal broadening of the excitation pulses and a variation of pulse arrival time across the FOV. To address this, we have characterised the consequent degradation of the spatio-temporal instrument response function (IRF) and mitigated this by incorporating a spatially varying temporal instrument response function in the FLIM analysis.

## 2. Methods

### 2.1 Experimental set-up

Our experimental design for the flexible endoscope follows previous work [23, 31, 32] collecting fluorescence with a gradient index lens (GRIN) objective lens bonded to a coherent fibre-optic bundle that relays it back to the detector. In some instruments the excitation radiation is also propagated through the fluorescence collection optical path but the commercially available coherent fibre-optic bundles typically present significant background fluorescence under u.v. or blue excitation [33]. We therefore chose to relay the excitation light through an additional optical fibre designed for u.v. transmission alongside the coherent fibre-optic imaging bundle. We have explored several strategies to provide a sufficiently uniform illumination over the 3 mm FOV provided by the GRIN objective lens with sufficient light efficiency to work with a few mW average power excitation sources. These have included combinations of single-mode or multimode optical fibres with microlenses or diffusers at the distal end. Utilising higher order modes in multimode optical fibres to increase the illumination FOV has the disadvantage that it leads to increased temporal dispersion of the excitation pulses and significant spatial variation in their arrival times at the sample but our experience has been that we cannot achieve sufficiently broad illumination and high light throughput with single mode optical fibres. To date, our best results with low excitation power sources have been obtained propagating the excitation radiation through a multimode optical fibre with a flat polished distal end face and no additional distal optical components. This simple design enables us to construct an endoscope of less than 2.3 mm in diameter such that it can be applied via the biopsy channel of many clinical endoscopes currently in use.

Figure 1 presents a schematic of this flexible wide-field endoscope system. The excitation source is either a gain-switched blue diode (PicoQuant, LDH-P-C-440M with driver PDL-800-B), which provides pulses of <500 ps at 40 MHz with average powers of ~3 mW, or a frequency doubled Ti:Sapphire laser (Newport Spectra-Physics broadband MaiTai) providing ~200 mW of ~100 fs pulses (subsequently broadened to ~100 ps by dispersion in the delivery fibre) at 80 MHz. This excitation radiation is filtered (F1, Semrock, FF02-438/24) to remove any unwanted

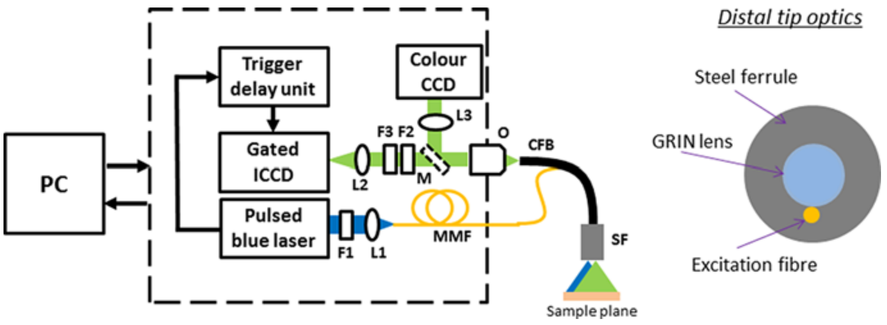

**Figure 1** Diagram of experimental set-up. Left: pulsed blue laser light from a diode is passed through a clean-up filter, F1 before being coupled into a multimode fibre (MMF) for delivery of excitation to the sample plane. A steel ferrule (SF) houses the excitation fibre tip alongside the GRIN lens and fibre-optic coherent imaging bundle. The fluorescence image is relayed via the coherent fibre-optic bundle (CFB) and imaged using a ×20 microscope objective (O) and tube lens (L2 or L3) to either a colour CCD camera or to the GOI (via interference and absorption filters, F2 and F3, used to block excitation light) using a switchable mirror (M). Right: diagram of en face view of distal probe tip showing the excitation fibre fixed in position beside the imaging optics.

excitation light that overlaps with the detection spectral window and coupled into a 3.5 m length of multimode fibre (FT400 UMT, Thorlabs, 0.39 NA, 200 μm core diameter) using an aspheric lens (A240TM-A, Thorlabs, 0.5 NA). The imaging path consists of a 1 mm diameter, 0.1 NA, 5 mm working distance GRIN objective lens (Grintech, GT-IFRL-100-005-50-CC) directly bonded to a 0.95 mm diameter, 2 m length of coherent optical fibre optic bundle with 30,000 cores (IGN-08/30, Sumitomo) using a UV curable epoxy (Norland, NOA61). Since each core in the bundle has a diameter of ~2 μm and a specified NA of >0.35, we expect propagation of the visible autofluorescence emission produced by blue excitation light to be multimodal.

At the distal end, a medical grade epoxy (Epotek, 301-2) is used to fix the multimode illumination optical fibre alongside the imaging optical fibre bundle in a protective steel ferrule (SF) of 2.3 mm outside diameter. The multimode excitation optical fibre was chosen such that the angular field of view of the imaging optics was matched by the distal-end cone of illumination obtained when light is coupled into all the modes of the fibre. This was realised by arranging the focusing of the beam onto the proximal end of the fibre to match the NA of the fibre. The large (~100,000) number of propagating modes superimpose to provide a smooth illumination profile. While this illumination strategy with the excitation fibre alongside the imaging lens is relatively simple to implement, its inherent weakness is that the imaged field of view and illuminated area do not completely overlap, resulting in a non-uniform illumination intensity, as is indicated in Figure 4(a). While it might be possible to mitigate this with more complex distal beam shaping optics, it is not a strong issue for FLIM because lifetime measurements are largely independent of excitation intensity. The variation in arrival time at the sample of the excitation pulses that propagated in different modes presents more of a challenge and this is addressed using a spatially varying IRF as described below.

At the proximal end the fluorescence is collimated by (O) a ×20 microscope objective (RMS20X, Thorlabs) and imaged onto the photocathode of the gated optical intensifier (GOI) by a 130 mm focal length achromat (L2) after passing through a 488 nm long pass interference (F2) (Semrock, BLP01-488R) and a 475 nm long pass absorption filter (F3). For FLIM, the pulsed excitation is synchronised with the GOI. A programmable rapidly-switchable delay generator (Kentech Instruments Ltd) is used to control the delay between the excitation pulse and the gating of the GOI which is coupled with standard camera lenses to a scientific cooled CCD camera (Orca-ER, Hamamatsu). The GOI is triggered to acquire a series of time-gated fluorescence intensity images at increasing delays with respect to the excitation pulses and thus the fluorescence decay profiles in each pixel of the FOV are sampled. The ORCA ER camera was operated with 4 × 4 hardware binning and a frame accumulation of 20 frames per delay time, resulting in a total acquisition time of less than 8 seconds for lifetime data acquired using 4 time delays. Post-acquisition, the images were further binned 2 × 2 in software, which improved the signal to noise without significantly impacting the resolution of the endoscope images. To acquire white light reflectance images for comparison with FLIM data, a 100% reflecting mirror (M) was used to manually switch the endoscope image from the GOI to a colour CCD camera (QImaging, Micro publisher 3.3). The apparatus and data acquisition were controlled using in house software (written in LabVIEW 7.1).

The spatial resolution of the wide-field endoscope was found to be ~25 line-pairs (lp)/mm by imaging a fluorescent USAF test chart (Newport, USAF-1951). This resolution is limited by the optical fibre bundle's pixelated structure, which has a core-to-core spacing of ~4 μm. This pixel sampling becomes 20 μm when the distal face of the fibre bundle is imaged onto the sample plane by the GRIN lens with ×5 magnification. The Nyquist sampling theorem therefore imposes a resolution limit of 25 lp/mm in the sample plane. We note that in the plane of the GOI photocathode, the image of the optical fibre bundle is magnified ×13 such that the core-to-core spacing becomes ~52 μm, which is adequately resolved by the GOI, which has a resolution of ~18 lp/mm.

## 2.2 Decay analysis

FLIM data was fitted to model fluorescence decay profiles using "*FLIMfit*", our custom-written open source software (http://www.openmicroscopy.org/site/products/partner/flimfit) [33]. For this work, the maximum likelihood estimator iterative fitting algorithm available in "*FLIMfit*" was used. In order to accurately measure fluorescence lifetimes, it is necessary to take into account any background contributions to the measured signal and the instrument response function, which is the response of the instrumentation to a delta function input. Here we must account for background fluorescence excited in the optical components of the endoscope, which we measure using a piece of black anodized metal placed in the sample plane and imaged under identical conditions for each data set (i.e. the same laser powers and acquisition times). This background is then subtracted from the measured FLIM data before fitting. The measured fluorescence decay profiles, $S(t)$, can be modelled as the convolution of the instrument response function, IRF($t$), and the sample's fluorescence response to a delta function exci-

Journal of
BIOPHOTONICS

tation pulse $F(t)$, i.e. $S(t) = \mathrm{IRF}(t) \cdot F(t)$ [1, 2]. Our FLIM detection scheme samples the fluorescence decay profiles in each pixel of the FOV and these measurements are then fitted to a model of the sample's fluorescence decay profile convolved with the IRF, which can be measured experimentally using a short lifetime dye or a scattered excitation pulse. The fitting of lifetime data is sensitive to the temporal profile of the IRF and the arrival time of the excitation pulses at the sample with respect to the triggering of the GOI.

In most FLIM instruments, the IRF is spatially invariant across the field of view and is generally defined by the temporal profile of the excitation pulse and the temporal resolution of the detector. With multimode optical fibre delivery of the excitation light, however, it is necessary to take account of the different propagation delay experienced by light propagating in different modes. Figure 2(a) illustrates schematically how modal dispersion in the excitation fibre leads to a spatially varying IRF. The temporal dispersion of meridional rays can be calculated using the following equation

$$\Delta t = \frac{Ln_0}{c}\left(\frac{1}{\cos\theta} - 1\right) \quad \text{for} \quad 0 \le \theta \le \theta_c$$

Figure 2(b) shows the expected dispersion of photon arrival times calculated for our FLIM endoscope of length $L = 3.5$ m, NA of 0.39 and core refractive index ($n_0$) of 1.46, which yields a critical ray angle of $\theta_c = 15.5^0$ inside and $\theta_{c\ air} = 23^0$ outside the optical fibre. Thus a systematic radial increase in excitation pulse arrival times up to ~700 ps is expected relative to the centre of the illumination profile at the sample plane, which is significant compared to the width of the IRF and to the fluorescence lifetimes measured.

## 2.3 Corrections for spatially varying IRFs

To be able to record accurate lifetimes using this FLIM endoscope, our fluorescence lifetime analysis software [34] was modified to incorporate spatially varying IRFs during iterative fitting of the model decay to the acquired data. Unfortunately, an IRF acquired by imaging a scattering sample cannot be used to directly measure the spatially varying IRF due to the difference in arrival times between scattered and fluorescence light caused by chromatic dispersion in the imaging fibre bundle. In principle, a short lifetime dye with similar excitation and emission wavelengths as the (tissue) samples considered in this work could be used but we were not able to identify a suitable dye. We therefore addressed this challenge by constructing a spatially varying IRF using a measurement of scattered excitation pulses to provide the temporal IRF profile to include in the fitting model but we adjusted its relative delay for each pixel to account for variations in arrival time of the excitation pulses. To obtain the required map of pixel arrival times, we imaged a solution of Coumarin 6, which presents a mono-exponential decay profile and similar spectral properties to the (tissue) samples of interest as shown in Figure 3. By cross-correlating the fluorescence decay profiles from every pixel in the image with that from a reference pixel ($x_r$, $y_r$), we could obtain a "relative delay map" ($\mathrm{Delay}_{\mathrm{relative}}$) of the IRF arrival times across the image with respect to the reference pixel. The global delay ($\mathrm{Delay}_{\mathrm{global}}$) between the Coumarin 6 reference pixel decay and the true IRF was then determined by fitting the Coumarin 6 fluorescence decay from the reference pixel using a scattered excitation light IRF ($\mathrm{IRF}_{\mathrm{scatter}}$) measured at the same reference pixel to determine the temporal offset. By combining the relative delay map and the global delay value, we construct a spatially varying IRF of the form

$$\mathrm{IRF}(t, x, y) = \mathrm{IRF}_{\mathrm{scatter}}(t - \mathrm{Delay}_{\mathrm{total}}(x, y), x_r, y_r)$$

$$\mathrm{Delay}_{\mathrm{total}}(x, y) = \mathrm{Delay}_{\mathrm{global}}(x_r, y_r) + \mathrm{Delay}_{\mathrm{relative}}(x, y)$$

This spatially varying IRF thus accounts for the effects of material dispersion and modal dispersion in the excitation optical fibre as well as dispersion in the imaging optical fibre bundle.

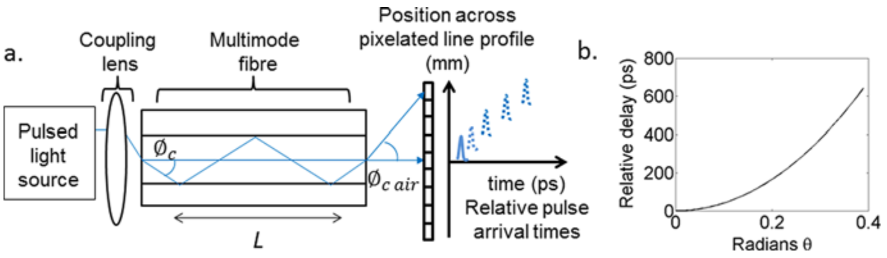

**Figure 2** (**a**) Schematic of rays propagating in multimode optical fibre with limiting (on-axis and meridional) modes indicated as rays, where $\theta_c$ is the critical angle for core/cladding interface; photons detected at different pixels can have different arrival times. (**b**) Relative delay of meridional rays relative to on axis rays for a 3.5 meter length 0.39 NA multimode fibre as a function of pitch angle $\theta$.

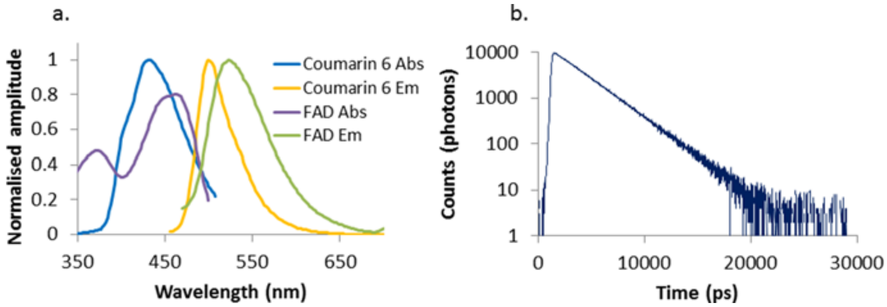

**Figure 3** (**a**) absorption (Abs) and emission (Em) spectra for Coumarin 6 and FAD (**b**) fluorescence decay profile of Coumarin 6 when excited by 445 nm laser light and detected at 490–600 nm (measured using TCSPC in a homebuilt fluorometer [35]).

### 2.4 *Rapid lifetime determination (RLD)*

The rapid lifetime determination (RLD) of fluorescence lifetime is an analytic method that calculates the lifetime, typically using the intensity measured for two or more parts of the decay profile (gates) [22, 36]. As such RLD requires only milliseconds of computation time to provide a fluorescence lifetime image and so can achieve faster FLIM update rates than iterative fitting techniques that typically require much longer computation times to converge. However, for complex fluorescence decay profiles, such as we expect from biological tissue, the lifetimes calculated using RLD represent a simple approximation, which is subject to ambiguity. It is therefore useful to provide rapid visual indications of difference between different (tissue) samples but cannot provide the quantitative information concerning the magnitude and relative contributions of different lifetime components that is available from iterative fitting to complex decay models or take account of systematic errors to the same extent.

### 2.5 *Human tissue specimens*

The study was approved by the Central London Research Ethics Committee (REC-1) (REC Ref 10/H0718/55) and conformed to the Declaration of Helsinki protocols. Patients scheduled for surgery at Northwick Park Hospital gave written informed consent for their tissue to be used in research.

### 2.6 *Mouse tissue*

ApcMin (Min, multiple intestinal neoplasia) mice were purchased from the Jackson Laboratory (Bar Harbor, ME) and bred in the animal facility at Imperial College London in a specific pathogen-free environment. Genotyping to identify mice that carry the mutant allele of the murine tumour suppressor Apc (adenomatous polyposis coli) was performed as previously described [37]. The intestine was washed with saline buffer to remove fecal material, opened longitudinally and the gut epithelium was exposed to image neoplastic polyps. Samples were kept moist using saline solution. Animal work was carried out in compliance with the British Home Office Animals Scientific Procedures Act 1986 (Project license number 70/7129).

## 3. Results

### 3.1 *Corrections for spatially varying IRFs: validation of method using fluorescent dye sample*

Figure 4 illustrates the validation of this approach by imaging a thin homogenous sheet of fluorescent dye (100 μM solution of Coumarin 6 dissolved in ethanol) confined between a pair of microscope coverslips. Figure 4(a) shows the time-integrated fluorescence intensity image of the dye sample, highlighting the spatial offset between the FOV of the collection optics and the illuminated area, as discussed in the experimental set-up section. Figure 4(b) shows the fluorescence lifetime map obtained when fitting the fluorescence data to a mono-exponential decay model with a uniform fixed IRF, illustrating how the variation in arrival time of the excitation pulses across the FOV produces errors. Figures 4(c), (d) show the fluorescence lifetime map and intensity-merged fluorescence lifetime image (i.e. the product of intensity image and lifetime map) obtained using the measured spatially varying IRF. Figure 4(e) shows the lifetime histograms corresponding to Figures 4(b), (c) with the spatially varying IRF-based analysis yielding a Gaussian shaped histogram, as expected when imaging a homogenous sample where Poisson noise is dominant. The mean fluorescence lifetime of ~2.5 ns is in agreement with our cuvette measurements of the same Coumarin 6 solution using time-correlated single photon counting (TCPSC).

Journal of BIOPHOTONICS

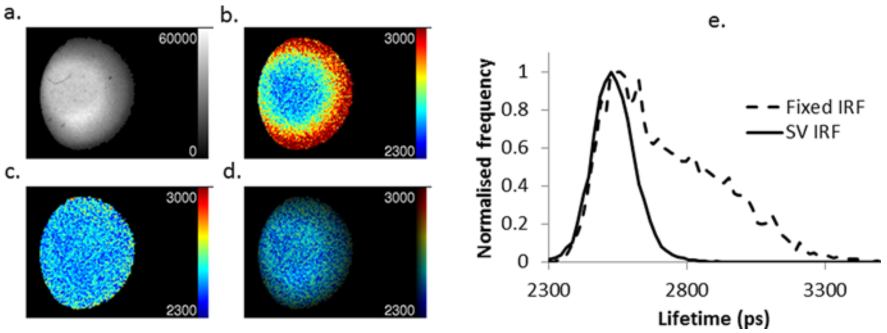

**Figure 4** Demonstration of the approach of using a spatially varying (SV) IRF to correct for variations in excitation pulse arrival time at the sample. (**a**) shows time-integrated fluorescence intensity image and (**b**) fluorescence lifetime image obtained from a thin layer of Coumarin 6; (**c**) fluorescence lifetime map and (**d**) intensity-merged fluorescence lifetime image obtained using a spatially varying IRF; (**e**) lifetime histograms obtained by iteratively fitting data obtained from a homogeneous Coumarin 6 dye sample to a mono-exponential decay model using a uniform and spatially varying IRF.

## *3.2 Application of FLIM endoscope to ex vivo imaging of diseased human laryngeal tissue*

Having established that we can utilise a single multimode excitation fibre to efficiently illuminate a 3 mm FOV, we studied the feasibility of using low cost gain-switched picosecond diodes for FLIM of tissue autofluorescence. For our first study, the FLIM endoscope system depicted in Figure 1 was mounted on a trolley of 71 × 79 × 120 cm with the excitation radiation provided at 445 nm by the ~3 mW average power gain-switched picosecond diode laser described above. This delivered ~0.5 mW to the sample plane to achieve irradiances of ~7 mW cm$^{-2}$, which was sufficient to realise FLIM with acquisition times of less than 8 seconds using 4 time-gate delays. This system was deployed at Northwick Park Hospital's Ear Nose and Throat department in a room next to an operating theatre so that human tissue samples could be imaged immediately following resection. Samples were kept moist using saline solution. Figure 5 shows reflected white light and fluorescence intensity and lifetime images of two samples of human larynx, together with histograms of the lifetimes obtained by iterative fitting to a mono-exponential decay model with the uniform fixed or spatially varying IRF.

Figures 5(a), (f) show reflected white light images of the specimens studied. Intense (white) specular reflections are due to surface fluid and paler regions of the tissue correspond to regions of lower blood content. Figures 5(b), (g) show time-integrated fluorescence intensity images and figures 5(c), (h) show intensity-merged lifetime images acquired using 4 time-gates of 1 ns duration separated by delays of 3 ns, for which the data were fitted to a mono-exponential decay model using the measured spatially varying IRF. Although tissue autofluorescence presents complex fluorescence decay profiles, mono-exponential analysis can still provide useful empirical contrast between different types or states of tissue. Data for these FLIM images took less than 8 seconds to acquire and the iterative fitting in *FLIMfit* took less than ~5 seconds. For each field of view imaged, three repeat measurements were made, which yielded a standard deviation in mean fluorescence lifetime between images of less than 10 picoseconds and indicated minimal photobleaching. To demonstrate the potential for faster imaging speeds, Figures 5(d), (h) show FLIM images calculated using *FLIMfit* with data from just two 2 time-gate delays (time-gates 1 and 3 selected from the 4 time-gate delays acquired in total), thereby demonstrating the potential to acquire wide-field endoscopic FLIM images in less than 4 seconds using irradiances of ~7 mW cm$^{-2}$ at the sample plane. These fluorescence lifetime maps are less accurate, presenting shorter lifetime values compared to the 4 time-gate measurements due to the under sampling of the longer decay components, but still provide useful lifetime contrast.

## *3.3 Ex vivo imaging of normal and neoplastic mouse bowel*

In order to demonstrate how this flexible wide-field FLIM endoscope could provide faster FLIM acquisition using higher power excitation diode lasers, we applied it to image a murine model of human colon cancer. The ApcMin mouse model recapitulates the disease developing in familial adenomatous polyposis (FAP) patients characterized by the presence of intestinal tumours and colon cancer due to a mutation in the Apc tumour suppressor gene. Bowel portions from Apc$^{min/+}$ mice were imaged and compared

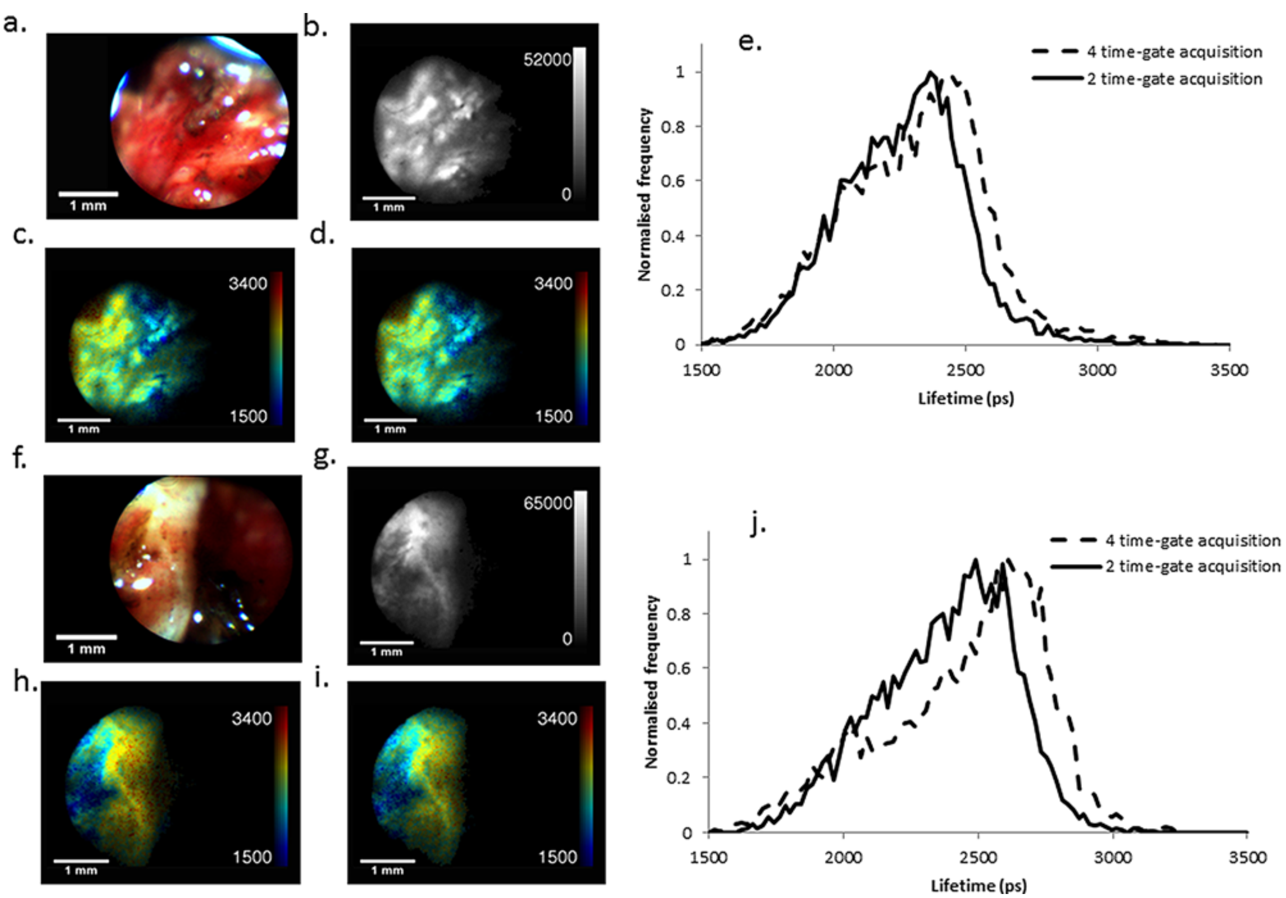

**Figure 5** FLIM endoscopy of diseased human larynx ex vivo. (**a**–**e**) and (**f**–**j**) correspond to two different diseased human larynx biopsies: (**a**, **f**) white light images, (**b**, **g**) time-integrated fluorescence intensity images; (**c**, **h**) 4 time-gate acquisition intensity merged FLIM images calculated using iterative fitting; (**d**, **i**) 2 time-gate acquisition intensity merged FLIM images calculated using iterative fitting; (**e**, **j**) histogram of lifetimes calculated from 4 or 2 time-gate delays.

to the control group of $Apc^{+/+}$ mice that do not develop tumours. We used a frequency doubled mode-locked Ti : Sapphire laser to provide excitation pulses at 445 nm. With 0.5 mW average excitation powers in the sample plane (equivalent to irradiances of ~7 mW $cm^{-2}$), acquisition times of less than 4 seconds were required for FLIM with 1 ns width time-gates at four delays spaced 3 ns apart.

Figure 6 shows a comparison of wide-field flexible FLIM endoscope images of normal $Apc^{+/+}$ (a, c, e, g) and diseased $Apc^{min/+}$ (b, d, f, h) mouse bowel tissue – the latter presenting a tumour, for which the autofluorescence lifetime shows clear contrast. Figures 6(a), (b) are reflected white light images recorded on the colour CCD camera and (c, d) are the corresponding time-integrated autofluorescence intensity images. Figures 6(e), (f) are FLIM images of the normal and diseased mouse tissue calculated by iteratively fitting data acquired at 4 time-gate delays to a mono-exponential decay model using the spatially varying IRF. Figures 6(g), (h) are FLIM images of the normal and diseased mouse tissue calculated from data acquired at only 2 time-gate delays (time-gate 1 and 3 selected from the 4 time-gate delays acquired) and fitted to a mono-exponential decay model using the spatially varying IRF. Figures 6(i), (j) show the corresponding lifetime histograms for the 4 time-gate FLIM and 2 time-gate FLIM, both of which show a visible mean lifetime shift for the diseased tissue in both the FLIM images and the histograms. In total 3 different fields of view were imaged at random locations across the normal and neoplastic epithelium from which mean lifetimes of $2.09 \pm 0.043$ ns and $2.211 \pm 0.049$ ns were obtained, suggesting there may be a difference in fluorescence lifetime between normal tissue and tumours that can be detected with the acquisition parameters chosen.

For live FLIM readouts required during in vivo imaging, a 2 time-gate sampling strategy can be combined with RLD. To this end we therefore increased the average excitation powers in the sample plane to 10 mW (equivalent to irradiances of ~7 mW $cm^{-2}$) and realised "real-time" fluorescence lifetime image update rates >2 Hz using RLD. No photobleaching

Journal of BIOPHOTONICS

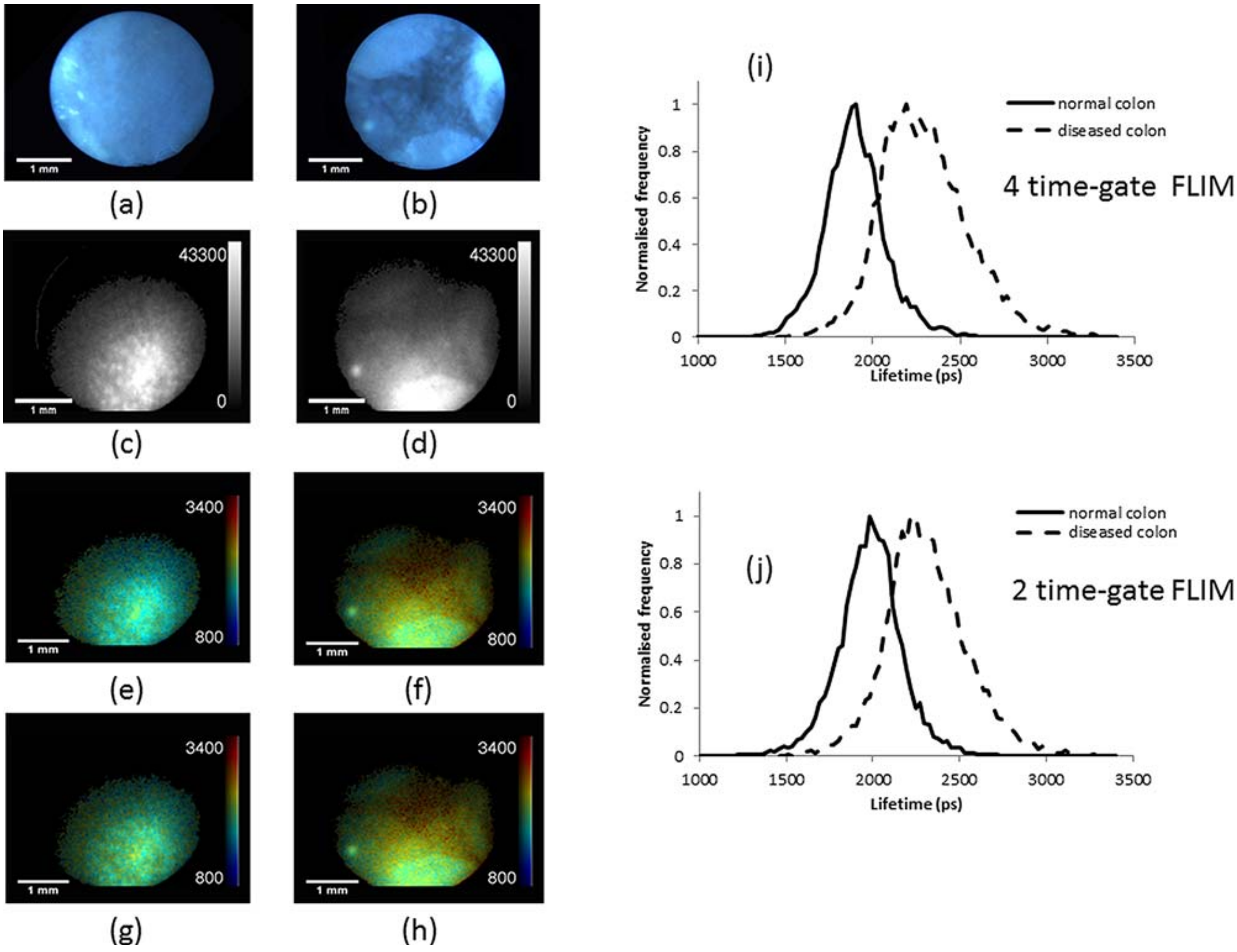

**Figure 6** (**a**) and (**b**): colour camera images of normal and neoplastic mouse bowel during white light reflectance imaging. (**c**) and (**d**): corresponding integrated fluorescence intensity images of the two fields of view of shown in (**a**) and (**b**). (**e**) and (**f**) 4 time-gate acquisition FLIM images using iterative fitting. (**g**) and (**h**) 2 time-gate acquisition FLIM images using iterative fitting; (**i**, **j**) corresponding lifetime histograms for the 4 time-gate FLIM and 2 time-gate FLIM.

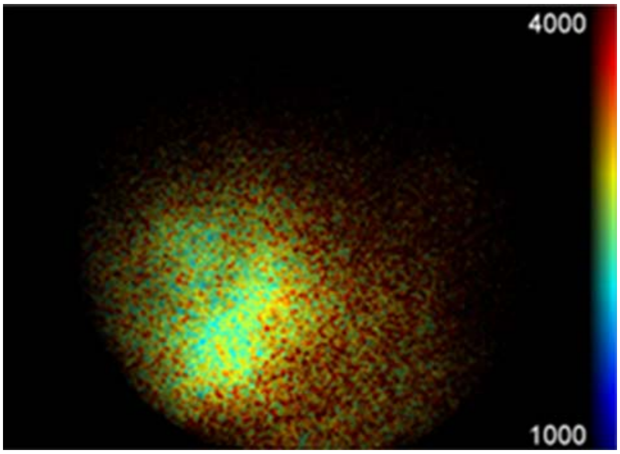

**Figure 7** Single frame from a ~2.5 Hz live update FLIM sequence of a neoplastic mouse bowel using RLD.

was observed for these irradiances. In Figure 7 we show a "real-time" FLIM endoscope image updated at 2.5 Hz and calculated using RLD from data acquired at two time-gate delays separated by 6 ns at which 4 frames were integrated per delay.

## 4. Conclusions

We have demonstrated the feasibility of flexible wide-field FLIM endoscopy utilising low average power blue excitation sources applied to tissue autofluorescence. This has been realised using a multimode optical fibre to efficiently deliver the excitation radiation to illuminate a 3 mm field of view. We have shown that the multimodal optical fibre propagation necessary to achieve this broad illumination can lead to errors in the lifetime determination unless the spatially varying IRF is taken into account. We have further demonstrated that this is practical, applying the FLIM endoscope to ex vivo tissue autofluorescence from human larynx and mouse bowel. The latter illustrates the potential for fluorescence lifetime imaging to screen for neoplasia using blue (445 nm) excitation in a flexible wide-field endoscope. The ex vivo human tissue FLIM data indicates the potential to develop new clinical instrumentation to aid diagnosis and monitoring of therapeutic interventions.

List of abbreviations
FLIM: Fluorescence lifetime imaging
FOV: Field of view
FAD: Flavo adenine dinucleotide
IRF: Instrument response function
GRIN: Gradient index lens
CCD: Charge coupled device
USAF: United States air Force
TCSPC: Time-correlated single photon counting
FAP: Familial adenomatous polyposis
RLD: Rapid lifetime determination

**Acknowledgements** This work has been supported in part by the UK Engineering and Physical Sciences Research Council (EPSRC, EP/F040202/1, EP/IO2770X/1) and the Wellcome Trust (RCDF088381/Z/09/Z). Hugh Sparks acknowledges an EPSRC-funded PhD studentship, Sean Warren acknowledges a studentship from the EPSRC-funded Institute of Chemical Biology Centre for Doctoral Training and Joana Guedes acknowledges an ISSF PhD studentship from the Wellcome Trust.

**Author biographies** Please see Supporting Information online.

## Supporting information

Author biographies
