# Supplementary material for: A flexible wide‐field FLIM endoscope utilising blue excitation light for label‐free contrast of tissue
Source: J Biophotonics. 2014 Feb 27;8(1-2):168–78. doi: 10.1002/jbio.201300203 (PMC4737404; doi:10.1002/jbio.201300203)
Supplement: Supplementary file 1 — Author biographies [file JBIO-8-168-s001.pdf]

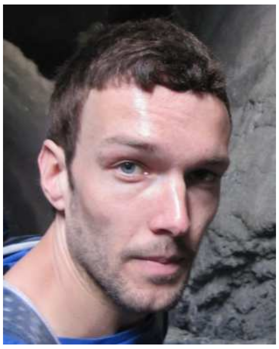

Hugh Sparks is a PhD student at the Photonics Group, Imperial College London developing novel endoscope techniques for biomedical applications. His research interests include FLIM of autofluorescence for label-free functional imaging of tissue and FLIM-FRET for monitoring protein interactions in vivo. He received his Masters in Physics from the University of Manchester in 2010.

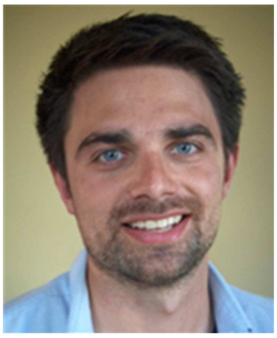

Sean Warren is a joint PhD student at the Photonics Group, Imperial College London and the Department of Structural and Molecular Biology, University College London developing multiplexed FLIM-FRET methodologies to study cell signalling pathways in chemotaxis. He developed FLIMfit as part of his PhD project. He received his undergraduate degree in Physics from the University of Cambridge in 2008 and a Master’s degree in Chemical Biology of Health & Disease from Imperial College London in 2010. He has previous worked as an Engineer at Roke

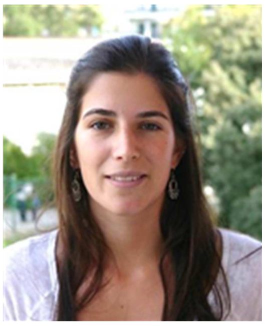

Joana Guedes received her MSc in Biological Engineering in 2012 from the Technical University of Lisbon in the *Instituto Superior Técnico* (UTL-IST). For her final year project she spent 6 months at University College London (UCL) in the Department of Biochemical Engineering. She is currently doing her PhD at Imperial College London in the Division of Cell and Molecular Biology. Her research interests focus on the role of natural killer (NK) cell receptors in liver inflammation and the detection of NK cells in the liver using intravital two-photon imaging

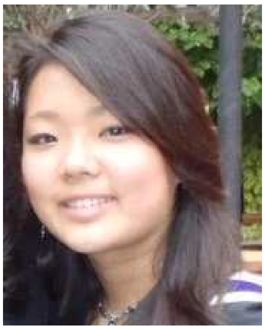

Nagisa Yoshida received her BSc in Biochemistry from Imperial College University, London 2012 and went on to receive her MRes in Biochemical Research in 2013 from the same university. Her interest in Immunology started during her Masters degree while working in Dr Nadia Guerra's group on intestinal tumorigenesis, where she continued to work as a research technician after her Master's in 2013. She is now following her research interests in Immunology as a research technician in Dr Eva Frickel's group at the MRC, National Institute of Medical Research (NIMR; Mill Hill, UK).

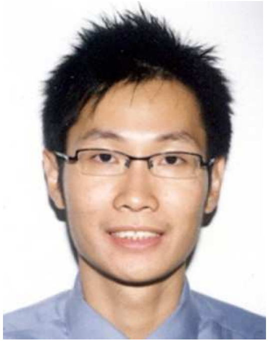

Tze Choong Charn completed his medical degree from the National University of Singapore in 2005. Subsequently he obtained post graduate qualifications of Member of the Royal College of Surgeons of Edinburgh (MRCS), Graduate Diploma in Family Medicine, Masters in Medicine (Otolaryngology), and Diploma of Otolaryngology – Head and Neck Surgery from 2006 to 2013. He is in his final year of Otolaryngology National Training Programme in Singapore.

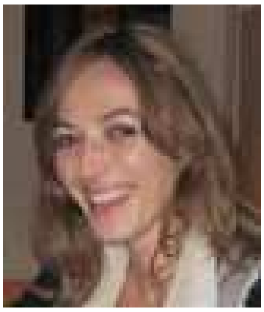

Dr. Nadia Guerra is a Lecturer in Immunology and Wellcome Trust research Fellow in the Department of Life Sciences at Imperial College. She has been working in the field of cancer biology and immunology for over 13 years. Dr. Guerra lab investigates the physiologic relevance of immunoreceptors expressed by Natural Killer (NK) cells in the development of immunopathologies and cancer. A multidisciplinary research program combining immunological approaches, genetic tools and 2-photons microscopy is used to investigate the dynamic and function of NK cells during inflammation and cancer.

Prior to that she completed her postdoctoral training with Pr. David Raulet at the University of California working with mouse models of cancer that mimic human disease. She made a significant contribution to the NK cell field through the generation of NKG2D-deficient mice, instrumental to the understanding of NK cell function in disease models.

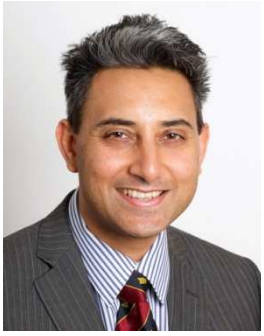

Taran Tatla received a First Class Honours Intercalated degree in Anatomy (1994) and gained M.B.,B.S. in 1996 whilst studying Medicine at University College London . Following completion of clinical studies he made rapid progress through highly competitive and comprehensive basic and higher surgical training programmes, incorporating posts at many of the renowned London teaching hospitals to be awarded FRCS in ORL-HNS in 2006. He then commenced his substantial NHS consultant post at North West London Hospitals NHS Trust, specializing in ENT disorders with Head & Neck sub-specialty interest.

He leads multi-disciplinary teams in investigating and treating patients with suspected voice and throat disorders, both benign and malignant, and has a particular interest in minimal access (endoscopic) and minimally invasive methods for disease management. He is presently pursuing a part-time PhD at Imperial College London in parallel with his clinical work, looking to develop

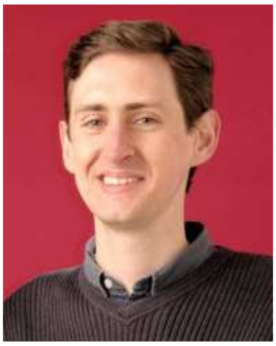

Chris Dunsby received an MSci. degree from Bristol University in 2000. In 2003 he received a Ph.D. from Imperial College, UK, in "Wide-field Coherence-gated Imaging Techniques Including Photorefractive Holography". He is now a joint lecturer between Photonics, Department of Physics and the Centre for Histopathology, Department of Medicine at Imperial. His research interests are centred on the application of photonics and ultrafast laser technology to biomedical imaging and include multiphoton microscopy, multiparameter fluorescence imaging and fluorescence lifetime

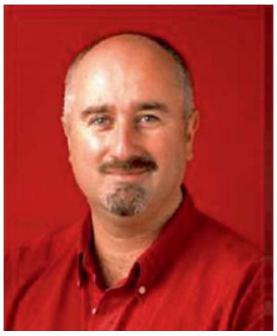

Paul M. W. French received the B.Sc. Degree in Physics in 1983 and the Ph.D. degree in 1987 from Imperial College London where he is currently a Professor in the Photonics Group of the Physics Department. He was a Royal Society University Research Fellow at Imperial from 1989 until 1994, when he joined the academic staff, and served as Head of the Photonics Group from 2001-2013. He also worked as a visiting professor at the University of New Mexico and a consultant at AT&T Bell Laboratories. His research has evolved from ultrafast dye and solid-state laser physics to biomedical optics with a particular emphasis on fluorescence lifetime imaging for applications in molecular cell biology, drug discovery and clinical diagnosis. Currently this includes the development and application of multidimensional fluorescence imaging technology for microscopy, endoscopy and tomography. He is a Fellow of the Institute of Physics, the European Physical Society and the Optical Society of America.
